# The injury severity score: an operations perspective

**DOI:** 10.1186/s12874-022-01528-6

**Published:** 2022-02-20

**Authors:** Nassim Dehouche

**Affiliations:** Mahidol University International College, Salaya, 73170 Thailand

**Keywords:** Injury severity score, Multicriteria decision making, Mutual information, Patient triage, Queuing theory

## Abstract

**Background:**

The statistical evaluation of aggregation functions for trauma grades, such as the Injury Severity Score (ISS), is largely based on measurements of their Pearson product-moment correlation with mortality. However, correlation analysis makes assumptions about the nature of the involved random variables (cardinality) and their relationship (linearity) that may not be applicable to ordinal scores such as the ISS. Moreover, using correlation as a sole evaluation criterion neglects the dynamic properties of these aggregation functions scores.

**Methods:**

We analyze the domain and ordinal properties of the ISS comparatively to arbitrary linear and cubic aggregation functions. Moreover, we investigate the axiomatic properties of the ISS as a multicriteria aggregation procedure. Finally, we use a queuing simulation with various empirical distributions of Abbreviated Injury Scale (AIS) grades reported in the literature, to evaluate the queuing performance of the three aggregation functions.

**Results:**

We show that the assumptions required for the computation of Pearson’s product-moment correlation coefficients are not applicable to the analysis of the association between the ISS and mortality. We suggest the use of Mutual Information, a information-theoretic statistic that is able to assess general dependence rather than a specialized, linear view based on curve-fitting. Using this metric on the same data set as the seminal study that introduced the ISS, we show that the sum of cubes conveys more information on mortality than the ISS. Moreover, we highlight some unintended, undesirable axiomatic properties of the ISS that can lead to bias in its use as a patient triage criterion. Lastly, our queuing simulation highlights the sensitivity of the queuing performance of different aggregation procedures to the underlying distribution of AIS grades among patients.

**Conclusions:**

Viewing the ISS, and other possible aggregation functions for multiple AIS scores, as mere operational indicators of the priority of care, rather than cardinal measures of the response of the human body to multiple injuries (as was conjectured in the seminal study introducing the ISS) offers a perspective for their construction and evaluation on more robust grounds than the correlation coefficient. In this regard, Mutual Information appears as a more appropriate measure for the study of the association between injury severity and mortality, and queuing simulations as an actionable way to adapt the choice of an aggregation function to the underlying distribution of AIS scores.

## Background

### Overview

The Injury Severity Score (ISS) is a widely-used aggregate indicator of the overall severity of multiple injuries to the human body that was introduced in a study by Baker et al. [1]. This score is calculated by summing the squares of the three highest values of the Abbreviated Injury Scale (AIS) [2], a common evaluation scale for the severity of trauma to individual body parts.

Since its introduction, the ISS plays an ambivalent role, which the present manuscript aims at discussing. It acts as both a clinical measure of the lethality of multiple injuries (Baker et al. [1] conjecture that this score “models a fundamental aspect of the human body’s response to multiple injuries”), as well as an operational indicator for patient triage. This ambivalence calls for two levels of analysis, when it comes to evaluating the ISS and similar aggregation procedures for AIS grades; a static study of their association with mortality and a dynamic evaluation of their axiomatic properties (i.e. how changes in AIS scores are reflected in the ISS) and queuing performance. However, only the former level of analysis is favored in the literature, with the correlation coefficient as sole association metric, and little is known about the axiomatic properties and queuing performance of ISS and similar aggregation functions.

### Original data source and results

The seminal study by Baker et al. [1] considered a sample of 2,128 motor vehicles occupants who were victims of accidents and admitted to one of 8 hospitals in the city of Baltimore, Maryland, USA, over a period of two years (1968-1969). For this sample, the study recorded a ratio of hospital admissions to deaths of 8:1. For individual hospitals, this ratio ranged from 5:1 to 60:1, indicating different levels of severity of injuries for the typical patient that each hospital received. Table 1 reproduces the distribution of AIS for the main injury of each patient in the sample, while Table 4 details the mortality rates corresponding to the highest AIS grade of patients in [1]. The authors find that the ISS explains 49% of the variance in mortality, in the study sample.

**Table 1:** Distribution of AIS grades over the sample of 2,128 patients in [1]

AIS Grade	Dead on arrival	Dead later	Survived	Unknown	Percentage
1	0	0	80	1	4%
2	0	2	437	1	20%
3	0	23	997	20	49%
4	0	30	229	3	13%
5	93	80	97	3	13%
Unknown	1	0	12	0	1%

### Construction of the ISS

The severity of damage to each of nine body regions (head, face, neck, thorax, abdomen, spine, upper extremities, lower extremities, and external) is conventionally evaluated on a scale of 0 to 5^1^ by the AIS. This scale evaluates individual injuries to a body region as follows:

0. No injury
1. Minor injury
2. Moderate injury
3. Serious injury
4. Severe injury
5. Critical injury

To compute the ISS, the nine previous body regions are first grouped into six:

- *R*_1_: Head or neck
- *R*_2_: Face
- *R*_3_: Chest
- *R*_4_: Abdominal or pelvic contents
- *R*_5_: Extremities or pelvic girdle
- *R*_6_: External

The ISS is then computed as the sum of the squares of AIS scores of the three most severe injuries, and is thus evaluated on a scale of 0 to 75.

Formally, let us denote *AIS*={*R*_1_,…,*R*_6_}, the AIS scores of an injured patient over the previous six body regions, which we will also refer to as the patient’s AIS profile. The computation of the ISS aggregates these score in two steps:

1. The three highest AIS scores, that is *A*= max(*AIS*),*B*= max(*AIS*−{*A*}), and *C*= max(*AIS*−{*A*,*B*}), are determined.
2. The sum of squares of *A*, *B*, and *C* is calculated, that is *ISS*=*A*^2^+*B*^2^+*C*^2^.

The first step of the ISS aggregation procedure (use of the three maxima) is justified in [1] by the fact that considering the sum of squares of the AIS scores of the three most severe injuries considerably improved the correlation of the resulting score with mortality rates, when including the fourth highest AIS score had no appreciable effect.

### Scope of this study

In this work, we will not analyze the first steps of the aggregation procedure and focus on the second. However, in The applicability of Pearson’s correlation to the ISS section, we show that statistical measures such as the correlation and standard deviation are not well suited for a variable such as the ISS, because they incorrectly assign it a cardinal value, which leads to inconsistent results. We should also mention an existing variant to the first step of the aggregation procedure, that questions not the use of three maxima for the AIS but the choice of body regions over which they are calculated. A widely-used such variant has been introduced under the denomination New Injury Severity Score (NISS) [3]. Instead, of considering the three most severely injured body regions, this variant considers the three most severe injuries overall, the reasoning being that the original ISS method can potentially disregard more severe injuries that happen to be in the same body region as the most severe injury. This medical modification is inconsequential to the analysis and claims made in this paper focusing on the intrinsic mathematical properties of the method. Our results apply to both variants.

Thus the main focus of this study is the second step of the aggregation procedure. Indeed, in [1] the choice of aggregating the three maxima by summing their squares was rather lightly justified as “the simplest nonlinear function”, without further explanations on the type of complexity being referred to. This justification will be put to question in the present work as the calculation of say the sum of cubes, or any other polynomial function of *A*, *B*, and *C* is no more complex than that of the ISS. As for the use of linear functions (e.g. summing the three maxima), it is dismissed in similarly vague terms with the sentence “the quantitative relationship of the AIS scores is not known and is almost certainly nonlinear”. The authors of the ISS further find that “the death rate for persons with two injuries of grades 4 and 3 was not comparable to that of persons with two injuries of grades 5 and 2 (sum = 7 in both cases)”.

After reviewing past work on the ISS, and notably the seminal study [1] that introduced this aggregation procedure, this paper questions the choice of a quadratic procedure relative to two other arbitrary aggregation functions (the sum and sum of cubes of the three highest AIS scores). Moreover, we study some axiomatic properties of the ISS and its queuing performance. Based on our results we propose that an injury severity aggregation procedure should be seen as an adjustment lever to optimize target criteria, rather than a rigid formula that seeks to capture fundamental aspects of the response of the human body to injuries with a quadratic formula (as has been wildly conjectured in the original study in the face of the high correlation of the ISS with mortality).

## Methods

### Measures of association between random variables

**Fig. 1:**
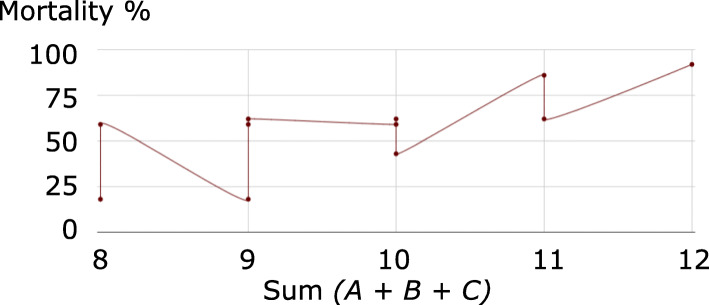
Mortality rates according to sum of the three highest AIS scores for the sample of 2,128 patients in [1]

**Fig. 2:**
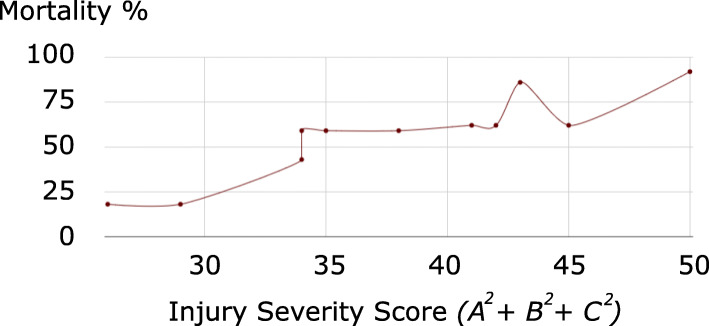
Mortality rates according to ISS (sum of squares of the three highest AIS scores) for the sample of 2,128 patients in [1]

**Fig. 3:**
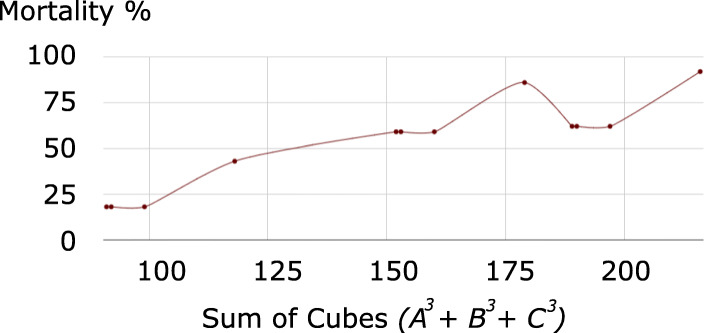
Mortality rates according to sum of cubes of the three highest AIS scores for the sample of 2,128 patients in [1]

For the study of the association between injury severity scores and mortality, measures of correlation with mortality are typically favored in the literature, and the (Pearson product-moment) correlation coefficient is typically used to evaluate the adequacy of ISS and competing proposals, as measurements of the lethality of injuries. However, the ordinal nature of the ISS and similar aggregation functions would naturally call for the use of rank correlation. Spearman’s rank-order correlation coefficient [4, 6] could be more appropriate measurements of the association between ISS and mortality rates. Indeed, this statistic evaluates the monotonic association between two variables without utilizing ordinal information. However, it cannot be precisely evaluated in the presence of ties, which are common as seen in Figs. 1, 2, and 3. Moreover, this indicator would be sensitive to the intrinsic variance of the ISS for consecutive values of the AIS, illustrated with the example in Table 5. A more robust measurement of the association between mortality and ISS would be offered by Mutual Information [7]. This more general indicator, which is less sensitive to the cardinal properties of random variables and is not limited to linear relationships, compares probability distributions as a whole and measures how different the joint probability distribution of two random variable is to the product of their marginal distributions. An extensive review and a general model for the use of mutual information for clinical decision making can be respectively found in [8] and [9].

Thus Mutual Information *MI*(*X*,*Y*), given by *MI*(*X*,*Y*)=*H*(*X*)−*H*(*X*|*Y*), between two random variables *X* and *Y* is the average amount of information (in bits) about one random variable that is gained by knowing the value of the other random variable. In this formula *H*(*X*) is the marginal entropy of *X*, given by $H(X)=-\sum \limits _{x \in D_{X}}p(x)\cdot log(p(x))$, and *H*(*X*|*Y*) the conditional entropy of *X* in regard to *Y*, given by $H(X|Y)=-\sum \limits _{x \in D_{X}, y \in D_{Y}} p(x,y)\cdot log \left (\frac {p(x,y)}{p(x)}\right)$, where *D*_*X*_ and *D*_*Y*_ are the respective support sets of *X* and *Y*, *p*(*x*,*y*) the joint probability distribution of *X* and *Y*, and *p*(*x*) the marginal probability distribution of *X*.

In its normalized form, mutual information quantifies this amount of information relative to the intrinsic entropy of each random variable. The normalized mutual information *NMI*(*X*,*Y*) between *X* and *Y* is thus given by $NMI(X,Y)=\frac {2\cdot MI(X,Y)}{H(X)+H(Y)}$. We compare the ISS with a linear and cubic aggregation functions, namely the sum and sum of cubes, using both Pearson’s correlation and Mutual Information.

### Axiomatic study

Little is known about the axiomatic properties and queuing performance of ISS and similar functions, including in the Operations Research literature. For the analysis of axiomatic properties, and given an AIS profile of the form (*A*,*B*,*C*), we introduce the notation [*x*_*A*_,*x*_*B*_,*x*_*C*_] such that −*A*≥*x*_*A*_≥6−*A*,−*B*≥*x*_*B*_≥6−*B*, and −*C*≥*x*_*C*_≥6−*C* indicate a change in the AIS profile of a patient (i.e. an overall degradation or improvement of their injuries), resulting in a new AIS profile (*A*+*x*_*A*_,*B*+*x*_*B*_,*C*+*x*_*C*_). We assume, without loss of generality, that these changes maintain the three most severe injuries located in the same three body regions (out of the six AIS body regions previously grouped). For instance, [−1,0,+1] represents an improvement of the most severe injury of a patient by one AIS point (e.g. following care), and a degradation of their third most severe injury by one AIS point, without any change to their second most severe injury. These vectors can be conventionally added with ISS profiles to obtain the resulting ISS profiles, e.g. a patient whose ISS profile is (4,3,2) would see their ISS profile become (4,3,2)+[−1,0,1]=(3,3,3), following the above described change. Using this notation, we study the axiomatic properties [10] of the ISS and test the compensation effects, rank reversals and independence property stemming from the use of the ISS as a multicriteria aggregation procedure.

### Queuing simulations

A queueing system is a general model of resource consumption, in which patients arrive at random times and require access to a healthcare resource (e.g. a physician consultation or inpatient bed). If the resource is busy upon a patient’s arrival, they are attributed a priority score and join a waiting line. In the present instance of the model, we consider the ISS, as well as the sum and sum of cubes of the three highest AIS scores as possible priority score. Other aggregation procedures, such as the NISS or the wISS could also be used, without loss of generality.

Defining a queueing model requires making stochastic assumptions about the nature of the arrival and service processes, as well as the distribution of AIS grades. In healthcare, the Poisson process has been verified to be a good representation of unscheduled arrivals to various healthcare units, including emergency departments [11].

The most common assumptions to make about arrivals and service times are the following:

- Arrivals follow a Poisson process characterized by a rate, that is the expected number of patient arrivals per unit of, denoted *λ*. The Poisson process for arrivals can also be conversely characterized by its expected inter-arrival time, that is the average time between two consecutive arrivals of patient, given by $\frac {1}{\lambda }$.
- The service rate is also described by a Poisson distribution with a mean service rate (i.e. number of patients served per unit of time) *μ*. This means that the service time for one customer follows an exponential distribution with an average of $\frac {1}{\mu }$.

The previous two assumptions are often called Markovian, and the resulting queuing model denoted M/M/s, where the two “M’s” stand for this adjective, and “s” for the number of identical service resources that customers queue to gain access to. For the sake of simplicity, we will assume the existence of a single resource, that is a so-called M/M/1 queue. An advantage of this model is that it only requires two parameters (*λ* and *μ*), which can be estimated empirically, in a fairly robust manner.

We conduct discrete-event simulations of an M/M/1 waiting line [11], with stochastic AIS grades, generated according to various distributions reported in the literature. This simulation allows us to study the queuing performance of the three aggregation procedures considered, as well as their sensitivity to the underlying distribution of AIS grades.

## Results

### On the use of a quadratic aggregation function

Table 2 describes the scales of the ISS (*A*^2^+*B*^2^+*C*^2^), as well as the sum (*A*+*B*+*C*) and sum of cubes (*A*^3^+*B*^3^+*C*^3^) functions. For *A*,*B*,*C*∈{0,1,2,3,4,5}, such that *A*≥*B*≥*C* and excluding triplet (0,0,0), there are 55 possible (*A*,*B*,*C*) triplets, resulting in 44 distinct possible values of the ISS (*A*^2^+*B*^2^+*C*^2^), as well as 13 and 55 distinct values of (*A*+*B*+*C*) and (*A*^3^+*B*^3^+*C*^3^), respectively.

**Table 2:** Possible scores and their rank, for the sum, sum of squares (ISS), and sum of cubes

Rank	*A*+*B*+*C*	*A*^2^+*B*^2^+*C*^2^	*A*^3^+*B*^3^+*C*^3^
1	1	1	1
2	2	2	2
3	3	3	3
4	4	4	8
5	5	5	9
6	6	6	10
7	7	8	16
8	8	9	17
9	9	10	24
10	10	11	27
11	11	12	28
12	12	13	29
13	13	14	35
14	14	16	36
15	15	17	43
16	-	18	54
17	-	19	55
18	-	20	62
19	-	21	64
20	-	22	65
21	-	24	66
22	-	25	72
23	-	26	73
24	-	27	80
25	-	29	81
26	-	30	91
27	-	32	92
28	-	33	99
29	-	34	118
30	-	35	125
31	-	36	126
32	-	38	127
33	-	41	128
34	-	42	129
35	-	43	133
36	-	45	134
37	-	48	136
38	-	50	141
39	-	51	152
40	-	54	153
41	-	57	155
42	-	59	160
43	-	66	179
44	-	75	189
45	-	-	190
46	-	-	192
47	-	-	197
48	-	-	216
49	-	-	250
50	-	-	251
51	-	-	253
52	-	-	258
53	-	-	277
54	-	-	314
55	-	-	375

We have computed all cases of discordance between the ISS, the sum, and the sum of cubes. In other words, the number of pairs of injury profiles for which the rankings provided by the two aggregation functions are reversed. Among the $C_{2}^{5}5=1485$ distinct, non-ordered pair of possible AIS profiles, we have identified the pairs for which there is discordance between *A*^2^+*B*^2^+*C*^2^,*A*^3^+*B*^3^+*C*^3^, and *A*+*B*+*C*, regarding the comparison of the pair. In other words, and for two patients *x* and *y*, let (*A*_*x*_,*B*_*x*_,*C*_*x*_) and (*A*_*y*_,*B*_*y*_,*C*_*y*_) be their respective AIS profiles. We consider that there is discordance between the ISS and the sum of cubes aggregation function if ($A^{2}_{x} + B^{2}_{x} + C^{2}_{x} > A^{2}_{y} + B^{2}_{y} + C^{2}_{y}$ and $A^{3}_{x} + B^{3}_{x} + C^{3}_{x} < A^{3}_{y} + B^{3}_{y} + C^{3}_{y}$) or ($A^{2}_{x} + B^{2}_{x} + C^{2}_{x} < A^{2}_{y} + B^{2}_{y} + C^{2}_{y}$ and $A^{3}_{x} + B^{3}_{x} + C^{3}_{x} > A^{3}_{y} + B^{3}_{y} + C^{3}_{y}$). There exist 84 pairs of profiles for which there is such a discordance, which represents 5.6*%* of the 1485 possible pairs of profiles (i.e. for a uniform distribution of AIS scores, the ISS and sum of cubes aggregation functions would disagree 5.6*%* of the time). The ISS and the sum are in discordance for 8% of possible profiles, whereas the sum of cubes and the sum are in discordance for 14.81*%* of possible profiles. Although a minority, these cases of discordance are non-neglectable, particularly for large volumes of patients.

#### Association with mortality

The seminal work [1] relied on the data in Table 3, which records the mortality rates for the AIS scores of the three most severe injuries, which we denote *A*, *B* and *C* by decreasing order of severity.

**Table 3:** Mortality by AIS scores of the three most severe injuries in [1]

Number of persons	102		78		38	
Most severe injury (*A*)	4		5		5	
Second most severe injury (*B*)	3		3		4	
Third most severe injury (*C*)	0-2	3	0-2	3	0-2	3
Percentage died	18%	43%	59%	86%	62%	92%

The use of the ISS was supported in [1] by the data reproduced in Table 4, in which we have additionally included the sums of the three most severe ISS, of their squares (the ISS), and of their cubes, and calculated the (Pearson product-moment) correlation and Mutual Information of each profile with mortality rates. Figures 1, 2, and 3 respectively plot mortality rates according to sum, sum of squares (ISS), and sum of cubes of the three highest AIS scores for the sample of 2,128 patients in [1].

**Table 4:** Mortality rates associated with the AIS profiles of the 2,128 patients in [1]

*A*	*B*	*C*	(ISS)			Mortality rate
			*A*+*B*+*C*	*A*^2^+*B*^2^+*C*^2^	*A*^3^+*B*^3^+*C*^3^	
4	3	0	7	25	91	18%
4	3	1	8	26	92	18%
4	3	2	9	29	99	18%
4	3	3	10	34	118	43%
5	3	0	8	34	152	59%
5	3	1	9	35	153	59%
5	3	2	10	38	160	59%
5	3	3	11	43	179	86%
5	4	0	9	41	189	62%
5	4	1	10	42	190	62%
5	4	2	11	45	197	62%
5	4	3	12	50	216	92%
Correlation with mortality			0.77	0.92	0.92	1.00
Mutual Information with mortality			0.46	0.55	0.71	1.00

The high (Pearson’s product-moment) correlation of the ISS and mortality has led [1] to conjecture that this score “models a fundamental aspect of the human body’s response to multiple injuries”. Though it remains a practical heuristic for priority evaluation and patient triage, the initial promise of the ISS as an indicator of the mortality of multiple injuries, and the even more daring conjecture of [1] that this quadratic function may capture fundamental properties of the response of human bodies to injuries have been tempered down by more mathematically rigorous, recent studies of the discrete possible values taken by the ISS. In [12], it has been found that mortality is non-monotonic with regards to the ISS, that is, mortality does not necessarily increases with successive values of ISS.

Following the same reasoning as [1], we use correlation with mortality as a measure of the adequacy of the three aggregation procedures. The sum of the three highest AIS scores presents the lowest correlation with mortality with 77% and Fig. 1 conveniently illustrates the reason for the inadequacy of this aggregation procedure. As indicated by the number of vertical and horizontal segments in the graph, the sum, which only offers 15 possible distinct values represented in Table 2, is not discriminant enough in relationship to mortality. However, the sum of squares (ISS) and the sum of cubes present similar levels of correlation with mortality rates, at 92% and as Figs. 2, and 3 show that the ISS (with 44 distinct possible value versus 55 for the sum of cubes, cf. Table 2) is less discriminant. All three functions are non-injective as evidenced by the existence of horizontal segments in the graphs. However, the relationship between the sum of cubes and mortality is of a functional nature (no vertical segments), as opposed to that of ISS with mortality. For instance, an ISS of 34 corresponds to both mortality rates of 43% and 59%. No such effects occur when considering the sum of cubes. However such undesirable effects cannot be evaluated by a coefficient of linear correlation, which would arbitrarily consider that the mortality rate associated with an ISS of 34 is 52%, the average of 43% and 59%.

#### The AIS and ISS are not cardinal measures

Measurement theory [13] assumes that there exist some empirical structure that one wishes to represent numerically (e.g. the body’s response to multiple injuries) and defines strict qualitative properties that the empirical structure must verify in order to be represented numerically. Such numerical artifacts are said to possess an interval level of measurement if, throughout its scale, equal differences in the measure reflect equal differences in the empirical structure being measured. Nothing indicates that the AIS and even less so the ISS possess such a property. The AIS and ISS can be more modestly considered to possess an ordinal level of measurement, that is to say as indicators allowing the ranking of patients, e.g. for triage purposes. An ordinal measure is defined, by opposition to a cardinal one, as “a variable whose attributes can only be ranked” [6, 14]. For instance, we know that an underlying injury having an AIS score of 3 is less severe than a 4, which in turn is less severe than a 5, but it remains unknown whether the distance between a 3 and a 4 is equal, greater, or smaller than the distance between a 4 and a 5. It is the practice of assigning the numerical values to the severity of these three injuries that sets the two numerical distances between them to be equal. The interpretation of the distances between ISS scores is similarly impossible. Indeed, the consecutive values in the domain of the ISS, represented in Table 2 only reflect an increase in the severity of the overall injury (ordinal information), but the extent of that increase cannot be given any interpretation (it contains no cardinal information). For instance, 50,51,54 are three consecutive values in the domain of the ISS, without any possible value between 51 and 54. A patient whose condition goes from an ISS of 50 to 51 and then from 51 to 54 would have seen the severity of their injury increase by two (ordinal) units, not four (cardinal) units.

Giving a cardinal meaning to the ISS could have been justified if the difference between two consecutive values of this scale kept increasing, reflecting a higher level of degradation as the severity of an injury increases, but this is not the case. In Table 2, we can observe for instance that the gap between the thirty-second and thirty-third grades of the ISS (scores of 38 and 41, respectively) is wider than between the thirty-fourth and thirty-fifth grades (scores of 42 and 43, respectively).

#### The applicability of Pearson’s correlation to the ISS

The value of the ISS is only ordinal, that is the information it provides is to rank the overall severity of injuries to multiple body regions of patients, and not measure any intrinsic property of these injuries. Further, [15] warns against considering the ISS/NISS as continuous statistical variables in correlation analyses with outcome measures (e.g. mortality), which has been the approach initially used to justify the quadratic aggregation of AIS grades in the original version of the ISS. If we accept the ISS as a purely ordinal indicator, a much simpler argument can be made to show that the very concept of measuring Person’s correlation of the ISS with any other variable does not apply. Pearson’s product-moment correlation is defined as the covariance of two variables divided by the product of their standard deviation [16]. Focusing on the ISS, we can observe that the concept of standard deviation does not apply to this variable.

Consider the toy example in Table 5 in which we measure the standard deviation of ISS, in three samples of two patients each. The two patients in each sample are of two consecutive ranks, with regards to the ISS (28*th* and 29*th*,32*nd* and 33*rd*, as well as 34*th* and 35*rd* ranks, respectively). Note that the ISS profiles of a patient in consecutive samples only differs by one unit of AIS (e.g. the three samples could correspond to a similar degradation of patient 1 and of patient 2 injuries over three periods of time).

**Table 5:** The variance of the ISS arbitrarily increases because of the uneven gaps between consecutive grades of the ISS scale

Sample	Patient 1	Patient 2	Patient 1 ISS	Patient 2 ISS	Variance of ISS
	ISS profile	ISS profile	(and rank)	(and rank)	in sample
A	(5,2,2)	(5,3,0)	33 (28th)	34 (29th)	0.5
B	(5,3,2)	(5,4,0)	38 (32nd)	41 (33rd)	4.5
C	(5,3,3)	(5,4,1)	42 (34th)	43 (35th)	0.5

We observe a significantly higher standard deviation and thus variance in sample B than in sample A, which is not due to a wider dispersion of the severity of injuries in sample B, but is solely due to the cardinal properties of the ISS. There happens to be no possible ISS values between 38 and 41. The range of ISS goes back to one unit in sample C, and we find the same variance as in sample A.

Thus, the very concept of a unit of deviation of the ISS is meaningless and no interpretation can be made of the standard deviation of this variable and hence of its covariance or Pearson correlation with any other variable. These concepts being based on that of a deviation of the observed ISS values relative to the mean, it is impossible to separate the amount of deviation that is due the observations and the amount due to the makings of ISS scale, with its uneven distances between grades.

The calculations of the standard deviation and variance of the ISS, as well as its Pearson’s correlation with mortality and the analysis of said correlation does not account for the average and standard deviation of the distance between two consecutive Injury Severity Scores (they are not one and zero respectively). It implicitly consider this score to be cardinal (i.e. a measure of the amount of something).

However for measures of mortality the average and standard deviation of the distance between two consecutive possible values are respectively one unit (depending on the decimal precision considered for mortality rates) and zero.

#### Mutual Information as a more appropriate measure of the association between injury severity and mortality

We have computed Mutual Information with the data in Table 4 as input, for the three considered aggregation procedures and with p-values of order of magnitude 10^−6^, we find normalized amounts of Mutual Information of 0.46, 0.55, and 0.71 between mortality rates in Table 4 and the sum, sum of squares, and sum of cubes of AIS scores, respectively. For this data-set, there is thus a significantly higher amount of information concerning mortality rates contained in the sum of cubes than the sum of squares, which confirms and quantifies the visual insight gained from Figs. 2 and 3 and suggests Mutual Information as a more appropriate measurement of the association between aggregate scores based on the AIS and mortality rates.

### Axiomatic properties

#### Arbitrary compensation

A multicriteria aggregation procedure is said to be compensatory if it allows for trade-offs between criteria, i.e. the possibility of compensating a disadvantage on some criteria by an advantage on other criteria [17]. The ISS being a simple sum of squares, it is a fully compensatory procedure, in that any disadvantage on any criterion (a lower AIS score) can be compensated by an advantage on any other criterion (a higher AIS score). For instance, improving the second most severe injury by one AIS point, while degrading the third most severe injury by two AIS points would bring the same change to the ISS, no matter its initial value.

Should a patient accept a medical procedure that improves your second most severe injury by one AIS point, but degrades your third most severe injury by two AIS points (for instance during transportation or waiting for said procedure)? Let us consider the toy example in Table 6.

**Table 6:** An improvement for Patient 2 (decrease in ISS) is a degradation for Patient 1 (increase in ISS)

Patient	Patient 1	Patient 2
Initial ISS Profile	(5,4,3)	(4,4,4)
Initial ISS	50	48
Change	[0,+1,−2]	[0,+1,−2]
Resulting ISS Profile	(5,5,1)	(5,4,2)
Resulting ISS	51	45

An improvement in Patient 2’s condition (decrease in ISS) is a degradation in Patient 1’s condition (increase in ISS).

This property of the ISS function is arbitrary. It does not have anything to do with the fact that Patient 1 was initially in a slightly worse state than Patient 2. It is due to the fact that trade-offs between AIS scores *A*, *b* and *C* in the calculation of the ISS do not obey a fixed compensation rate. The very notion of improvement or degradation of the AIS score is thus meaningless. It should be noted that weighted aggregation procedures, such as the recently introduced weighted ISS (wISS) by Shi et al. [18] do not suffer from this inconsistency, as the trade-off rates between criteria would be constant and defined by their weights.

#### Arbitrary rank reversals for identical changes

Table 7 shows a toy example in which Patient 1 and Patient 2 receive twice the same procedure (an improvement of their most severe injury by one AIS point followed by an improvement of their second most severe injury by one AIS point). Initially, the overall condition of Patient 2 (ISS of 33) is worse than that of Patient 1 (ISS of 32). However, after the first procedure the order of severity of the conditions of the two patients alternates to Patient 1 (ISS of 25) being worse off than patient 2 (ISS of 24) and then back to Patient 2 (ISS of 21) being in a worse condition than Patient 1 (ISS of 20), after the second procedure. Moreover, Table 8 shows a similar alternation of priority but with the condition of the two patients progressively degrading over time). In a situation where the ISS is used as a triage rule, the order of priority between the two patients would arbitrarily alternate, although the degradation of their states would be identical.

**Table 7:** The order of priority of the two patients arbitrarily alternates despite an identical improvement of one of their AIS grades

Patient	Patient 1	Patient 2
Initial ISS Profile	(4,4,0)	(5,2,2)
Initial ISS	32	33
Change	[−1,0,0]	[−1,0,0]
Resulting ISS Profile	(4,3,0)	(4,2,2)
Resulting ISS	25	24
Change	[0,−1,0]	[0,−1,0]
Resulting ISS Profile	(4,2,0)	(4,2,1)
Resulting ISS	20	21

**Table 8:** The order of priority of the two patients arbitrarily alternates despite an identical degradation of one of their AIS grades

Patient	Patient 1	Patient 2
Initial ISS Profile	(4,4,0)	(5,2,2)
Initial ISS	32	33
Change	[0,+1,0]	[0,+1,0]
Resulting ISS Profile	(5,4,0)	(5,3,2)
Resulting ISS	41	38
Change	[0,0,+1]	[0,0,+1]
Resulting ISS Profile	(5,4,1)	(5,3,3)
Resulting ISS	42	43

#### Independence

The independence property states that identical performance on one or more criteria should not influence the way two alternatives compare [10]. A transformation that maintains the value of the criterion equal should not change the way alternatives compare. In Table 9, we consider two pairs of ISS profiles, Patient 1 and Patient 2 versus Patient 3 and Patient 4. The only difference between these two pairs concerns the AIS score of the most severe injury (3 and 4 for patient 1 and patient 2 respectively, 4 and 5 for patient 3 and patient 4 respectively). An identical change, [0,+1,0]. is applied twice to the second most severe; it gains one point of severity. The two pairs of patients show an identical level of severity, in their second and third most severe injuries before and after the transformation, respectively (.,2,0) and (.,0,0). However, the change leads to two different outcomes. Patient 1 condition (*ISS*=13), which was initially less severe than that of Patient 2 (*ISS*=16), becomes more severe (18>17), whereas the order of priority of Patient 3 and Patient 4 remain unchanged (20<25 and 25<26).

**Table 9:** An identical change to the second most severe injury *ceteris paribus* leads to different outcomes

Patient	Patient 1	Patient 2	Patient 3	Patient 4
Initial ISS Profile	(3,2,0)	(4,0,0)	(4,2,0)	(5,0,0)
Initial ISS	13	16	20	25
Change	[0,+1,0]	[0,+1,0]	[0,+1,0]	[0,+1,0]
Resulting ISS Profile	(3,3,0)	(4,1,0)	(4,3,0)	(5,1,0)
Resulting ISS	18	17	25	26

### Queuing simulation

#### Settings

Viewing AIS aggregation procedures, such as the ISS, as priority indicators for access to healthcare resources, rather than fundamental measures of the body’s response to multiple injuries, one can focus on evaluating their operational performance. Queuing theory is an important tool in the Operations Research toolset with fruitful applications in healthcare, a systematic review of which can be found in [19]. It can offer valuable insights on the dynamic properties of triage rules, when deployed for large-scale patient flows, and help inform the choice of an appropriate priority regime. However, to the best of our knowledge, little is known in the literature about the queuing performance of the ISS and similar trauma indicators. This section proposes a model for their evaluation, based on a discrete-event simulation of a M/M/1 queuing system.

Throughout the present simulation, we consider an average service time of *μ*=1. In other words, we take one time-unit to represent the average service time of a patient. For instance, if the resource under study is a hospital bed, and the average length of stay is one week, one unit of simulation time would correspond to one week. If, on the other hand, it is access to a physician, with an average consultation duration of ten minutes, one unit of simulation time would correspond to ten minutes. Moreover, and in order to create a congested waiting line, we consider an average inter-arrival time of $\frac {1}{\lambda }=0.1$, meaning that, on average, ten patients arrive in the queue during the time it takes to deliver the service to one patient.

Since the focus of this study is on priority regimes and their impact on queuing performance, we additionally need to make assumptions regarding the distribution of AIS grades of arriving patients. We have conducted our simulations with respect to different distributions of AIS grades reported in the literature. In addition to the distribution for victims of motor vehicles accidents, reported by Baker et al. [1] and reproduced in Table 1, we consider the distributions of AIS grades for 174 adult victims of fall accidents reported by Lopes et al. in [21], 451 patients with tornado-related injuries reported by Deng et al. in [22]^2^, and 278 victims of traumatic maternal injuries reported by Awoleke et al. in [23]. The details of each distribution are reproduced in Table 10 and the code in Appendix A.

**Table 10:** Distributions of AIS grades reported in five select studies

Data source	AIS=1	AIS=2	AIS=3	AIS=4	AIS=5
Baker et al., 1974 [1]	42%	20.2*%*	4.92*%*	13.2*%*	13.2*%*
Lopes et al., 2014 [21]	36.57*%*	29.73*%*	27.81*%*	3.54*%*	2.32*%*
Deng et al., 2018 [22]	60.85*%*	15.46*%*	14.46*%*	6.73*%*	17.5*%*
Awoleke et al., 2019 [23]	40.06*%*	48.6*%*	8.30*%*	25%	0%

For the three aggregation procedures considered in this study (ISS, sum and sum of cubes), we are interested in evaluating discrepancies in the average waiting time for all patients and for patients with critical injuries (i.e. patients presenting AIS scores of 5 on some body regions), as a proxy for mortality. These discrepancies would result from the cases of discordance between the three aggregation procedures, discussed in On the use of a quadratic aggregation function section.

For each distribution of AIS grades in Table 10, we conduct 100 simulation, each simulation having a duration of 1000 discrete time-units. We estimate the average waiting times per patient, resulting from each of the three aggregation procedures. The commented source code for these simulations and their evaluation is provided in the R language, in Appendix A.

#### Results

**Fig. 4:**
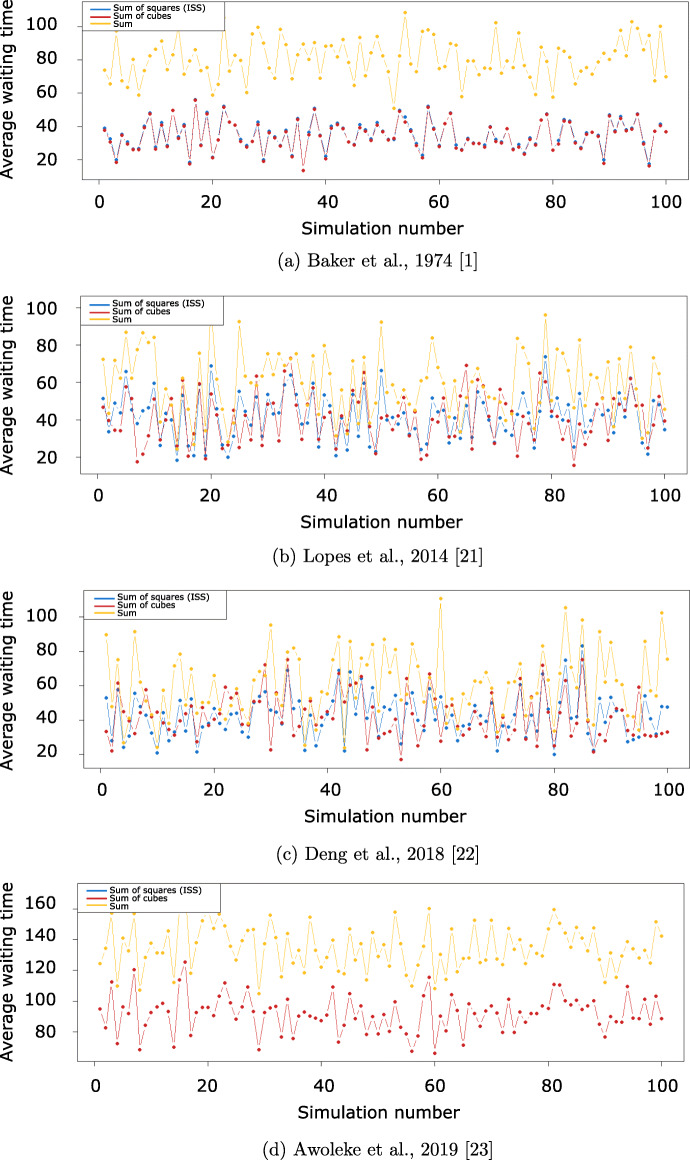
Average waiting time per patient, for 100 simulations with different AIS distributions. One time-unit corresponds to the average service time of a patient

**Fig. 5:**
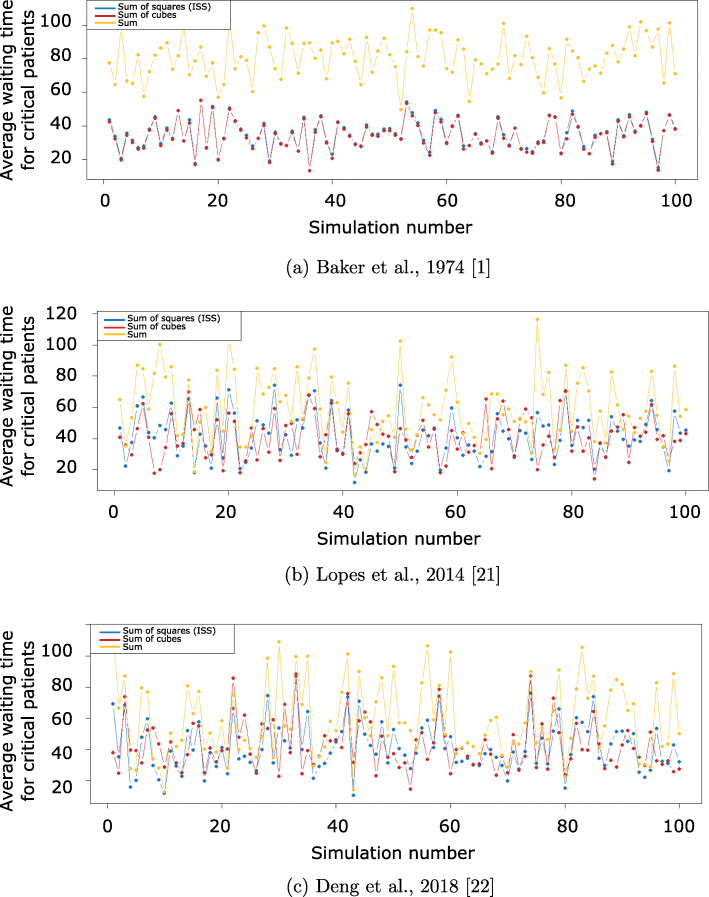
Average waiting time per critical patient, for 100 simulations with different AIS distributions. One time-unit corresponds to the average service time of a patient

Figures 4 and 5 respectively detail the average waiting times for all patients and critical patients, in each simulation, for the four AIS distributions considered, while Table 11 presents their averages over the 100 simulations.

**Table 11:** Overall average waiting times for all patients and critical patients, over 100 simulations, where *A*, *B*, and *C* are the three highest AIS grades, in decreasing order

Data source	Average waiting time for all patients			Average waiting time for critical patients		
	*A*+*B*+*C*	*A*^2^+*B*^2^+*C*^2^	*A*^3^+*B*^3^+*C*^3^	*A*+*B*+*C*	*A*^2^+*B*^2^+*C*^2^	*A*^3^+*B*^3^+*C*^3^
Baker et al., 1974 [1]	71.48	39.14	38.49	71.47	39.12	38.45
Lopes et al., 2014 [21]	61.11	41.58	40.31	61.80	42.03	39.17
Deng et al., 2018 [22]	72.56	42.71	42.42	58.89	42.39	42.15
Awoleke et al., 2019 [23]	134.90	91.93	91.93	N/A	N/A	N/A

It should be noted that, since the AIS distribution reported by Awoleke et al. [23] does not include any critical patients, this distribution is excluded from the computation of waiting times of critical patients. As per the setup of these simulations, the time-unit of average waiting times corresponds to the service time. For instance, if the resource under study is a hospital bed, with average length of stay of one week, an average waiting time of 39.14 would correspond to 39.14 weeks. If, on the other hand, it is access to a physician, with an average consultation duration of ten minutes, it would correspond to an average waiting time of 391.4 minutes.

These simulations confirm the inefficiency of the sum as an aggregation procedure. Indeed, it results in significantly longer average waiting times, and only outperforms the ISS in some rare simulations. However, the comparison of queuing performance is more nuanced between the ISS and the sum of cubes. The two aggregation procedures show identical performance for the AIS distribution of Awoleke et al. [23], which can be explained by the relatively lower AIS scores in this distribution, and the fact that cases of discordance between the ISS and the sum of cubes (14.81*%* of possible AIS profiles, as discussed in On the use of a quadratic aggregation function section) mainly occur for higher AIS values. However, on average over the 100 simulations, there is a non-negligible advantage to using the sum of cubes, in terms of minimizing average waiting for all patients and critical patients alike. For the distribution of Lopes et al. [21], this advantage is as significant as 2.86 units of time, on average, for critical patients. This advantage can be explained by the that the sum of cubes offers a broader set of possible scores than the ISS (55 vs 41, as shown in Table 2), thus allowing it to convey more information. This fact was also reflected in its higher mutual information with regard to mortality in Table 4. However, these results should not be interpreted as the sum of cubes being a universally better aggregation procedure than the ISS, as these simulations were only conducted under specific simplifying assumptions and for a select set of AIS distribution. For different queuing settings and empirical AIS distributions, the ISS may very well be the best performing aggregation procedure. Indeed, the most general and robust conclusion we can draw from the results of these simulation is that the operational performance of an aggregation procedure is sensitive to the underlying AIS distribution and thus the choice of the “best” procedure can only be made on a case-by-case basis, with respect to empirical estimates of this distribution in a healthcare unit.

## Discussion

Aggregation procedures for AIS grades, such as the Injury Severity Score and similar, competing indicators (New Injury Severity Score [3], Exponential Severity Score [20], etc.) have important operational applications as waiting line priority regimes. Therefore, their design is a highly sensible one that impacts mortality rates. However, the evaluation of these indicators typically relies on a static, linear evaluation of their association with mortality rates, and proposals typically compete on which function achieves the highest Pearson correlation. In this paper, we put forward the idea that curve-fitting and the evaluation of correlation with mortality rates are insufficient evaluation methodologies for the operational performance of these aggregation procedures. We have shown correlation-based measurements (as well as measurements of the standard deviation/variance of the ISS) to be largely unfounded, and proposed Mutual Information as a more adequate and more general measure of association. Moreover, by attempting to be two things at once (a cardinal measure of the human body’s response to multiple injuries as well as an ordinal triage rule presenting good association with mortality), the ISS may achieve sub-optimal results in both regards. A complex, fundamental property such as the physiological response to injury is unlikely to be universally captured by a simple mathematical function (the ISS) of ordinal mathematical measures (the AIS). Thus, there can be no universally best aggregation function. We recommend viewing the ISS, and similar aggregation procedures for multiple AIS grades, as purely operational triage indicators, rather than cardinal measures of the response of the human body to multiple injuries. As such, the choice of such an aggregation function should be made according to the distribution of AIS grades in a healthcare unit, to optimize queuing performance.

## Conclusions

The present paper studied the Injury Severity Score as a multicriteria aggregation procedure for operational decision-making. We have highlighted some of its statistical and axiomatic properties that can lead to bias in its large-scale usage as a patient triage indicator. These properties therefore present areas of improvement for future proposals of aggregation procedures. Moreover, and although the addition of a degree to this quadratic aggregation procedure (i.e. considering the sum of cubes rather than the sum of squares) was found to convey more information on mortality and improve waiting line performance, the ISS was generally found to be a robust triage rule that achieved decent waiting line performance. However, we have shown this performance to be highly sensitive to the statistical distribution of the AIS scores of patients entering the waiting line. Thus, these findings suggest that the choice of an aggregation procedure for AIS grades (ISS, sum of cubes, or any other function) should be made on a case by case basis, with respect to the empirical distribution of these grades in a trauma department. This perspective notably permits the design of aggregation procedures for AIS grades in a way that explicitly optimizes operational criteria, such as the average waiting time of patients presenting critical injuries. In our view, the ambiguous, classical view in the literature of the ISS as a cardinal measure of the severity of multiple injuries (besides its use as an ordinal triage indicator) and the ensuing correlation analyses with mortality rates have somehow hindered this actionable line of research.

## Appendix A

### R Script for queuing simulation


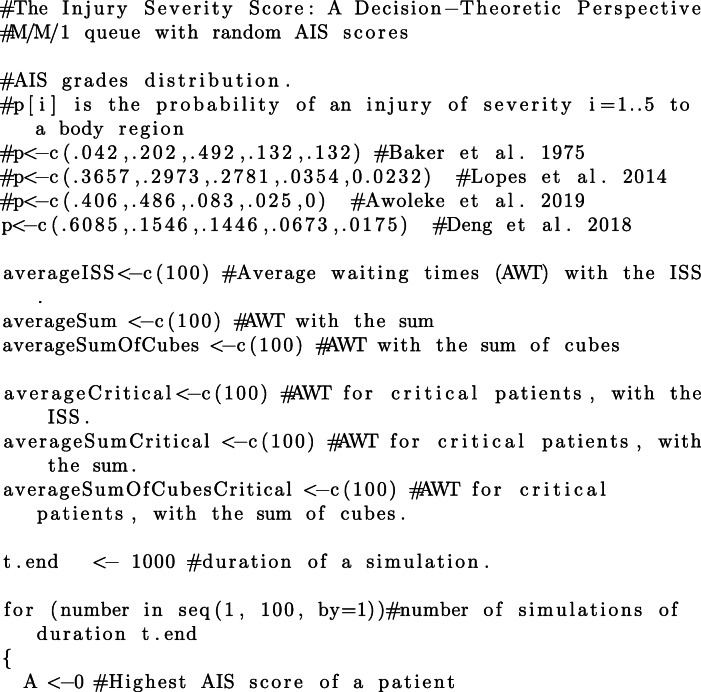



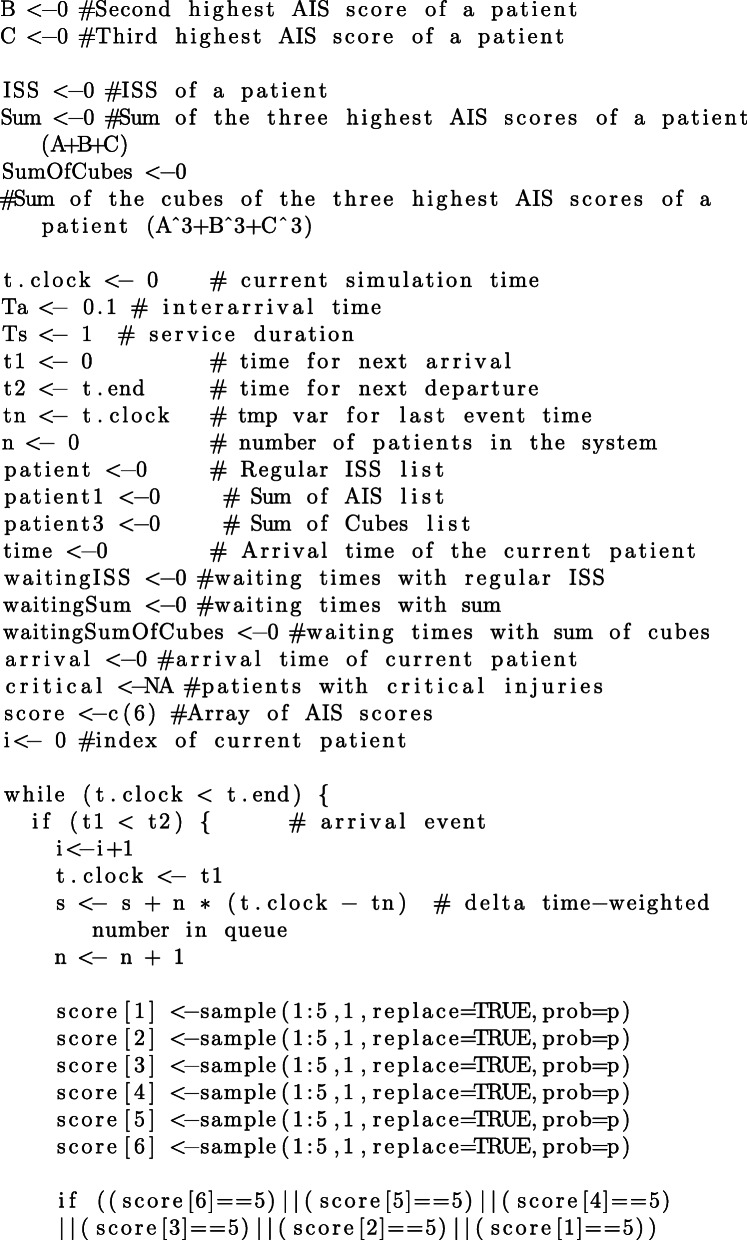



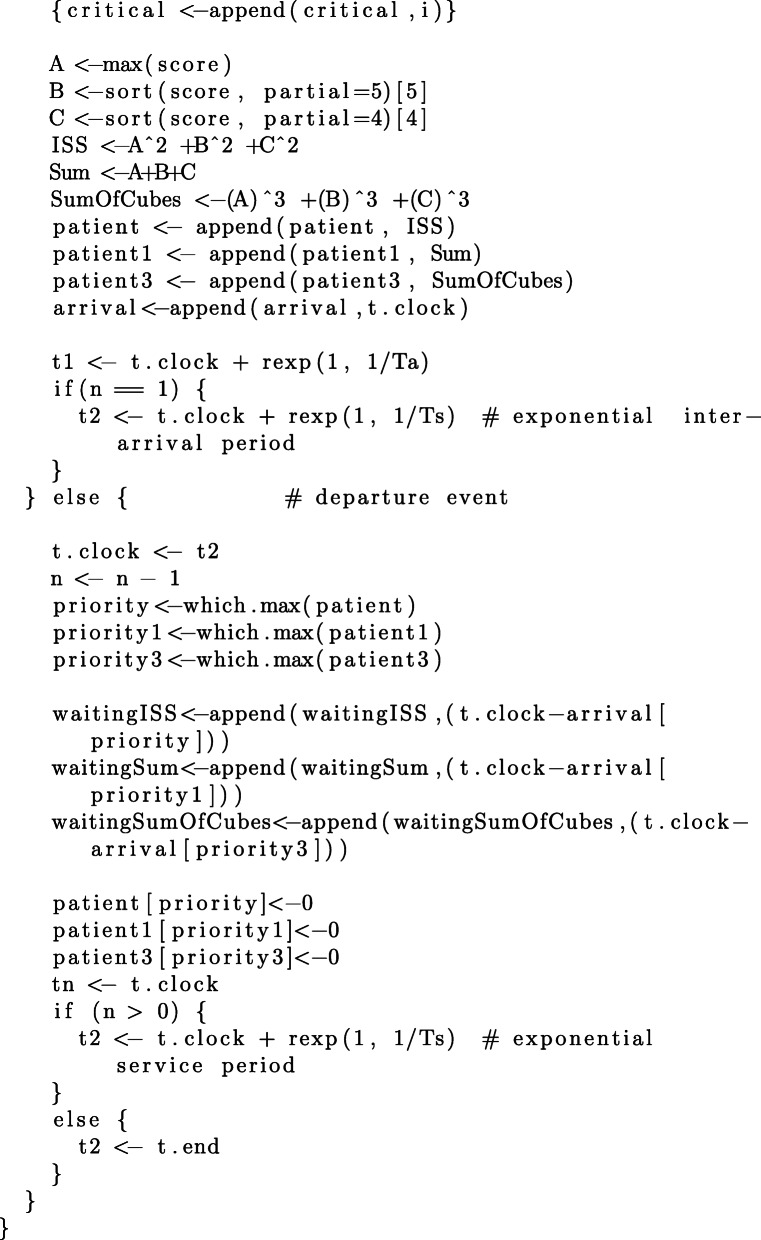



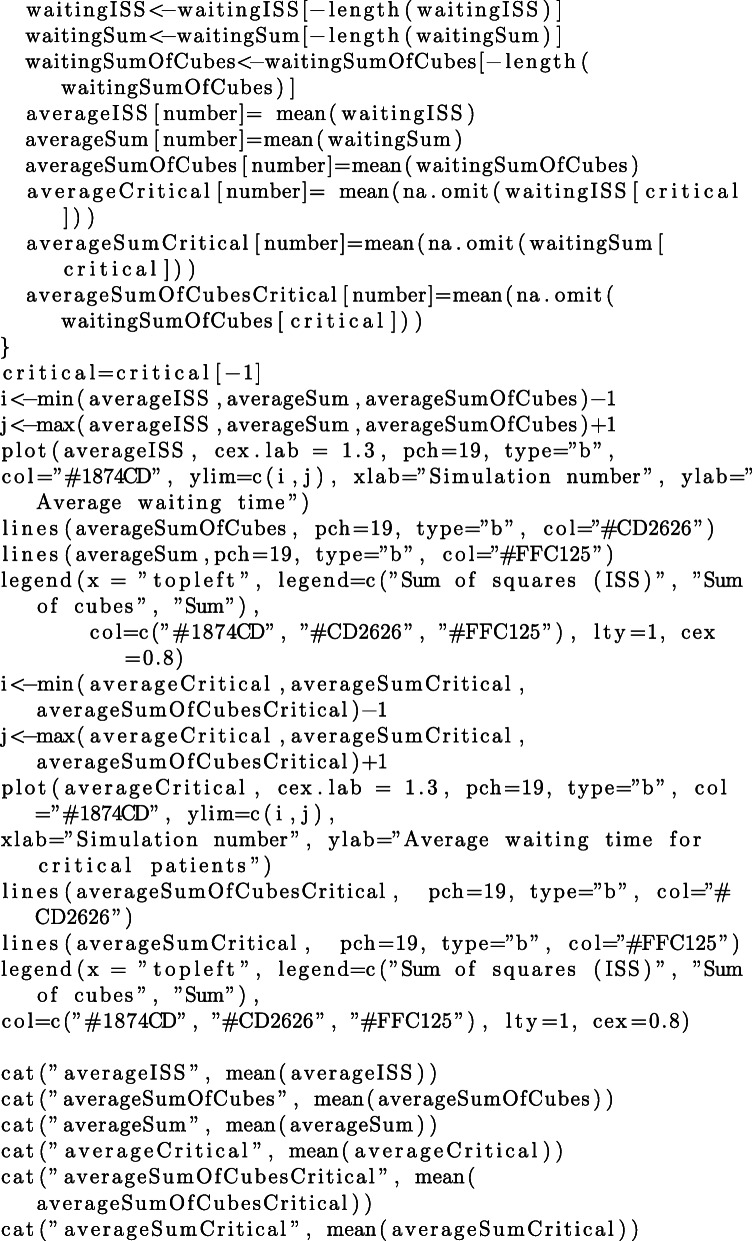


## Data Availability

The data used in the current study are available in references [1], [21], [22], and [23]. The source code for the queuing simulation is provided in the R language, in Appendix A.

## Abbreviations

AIS: Abbreviated injury scale
ISS: Injury severity score
NISS: New injury severity score

## Footnotes

1 A grade of 6 additionally indicates untreatable injuries. This value being immaterial to the purpose of this paper, we will omit it from our analysis

2 This study also included 0.75*%* of cases presenting fatal injuries (AIS=6), this grade being immaterial to the purpose of the waiting line simulation conducted herein.
